# Too Many Treats or Not Enough to Eat? The Impact of Custodial Grandparents on Food Security and Nutrition

**DOI:** 10.1093/geroni/igaa057.1125

**Published:** 2020-12-16

**Authors:** Danielle Nadorff, Rahel Mathews

**Affiliations:** Mississippi State University, Mississippi State, Mississippi, United States

## Abstract

In the US, 28.5% of seniors are obese, with a BMI of 30 plus. The prevalence of obesity for children is also an alarming 17%, making it one of the primary public health burdens. According to the socio-ecological model, a child’s weight status can be influenced by factors related to parenting style, family, and the community. The literature reflects a significant emphasis focusing on children and their parents. However, according to the US Census, 7.5 million grandchildren are living with their grandparents, with about 1/3 of these residing in skipped-generation households. There are essential age-related differences in food preparation and eating behaviors between middle-aged and older grandparents and younger adult parents that may influence their children’s eating behaviors. Grandparents may provide a positive feeding environment, including role-modeling healthy food intake, teaching children about nutrition, and involving them in mealtimes and cooking, monitoring and encouraging children to eat nutritious foods, especially vegetables and regularly serving vegetables. However, grandparents have also reported providing energy-dense and nutrient-poor food and drinks and used food as a reward or gift. The current study aims to investigate the influence of caregiver type (grandparents only, parents only, or multigenerational households) on children’s nutrition, food security, and BMI. One-way ANCOVAs controlling for SES found that grandparent-headed households had children with more deficient diet and higher BMIs, but also less food insecurity. These results indicate that age-related changes in caregiver type are an important predictor of children’s nutritional health. Details and clinical implications will be discussed.

